# A Simple and Selective Fluorescent Sensor Chip for Indole-3-Butyric Acid in Mung Bean Sprouts Based on Molecularly Imprinted Polymer Coatings

**DOI:** 10.3390/s17091954

**Published:** 2017-08-24

**Authors:** Jiahua Chang, Bota Bahethan, Turghun Muhammad, Burabiye Yakup, Mamatimin Abbas

**Affiliations:** 1 Key Laboratory of Oil and Gas Fine Chemicals, Ministry of Education & Xinjiang Uygur Autonomous Region, College of Chemistry and Chemical Engineering, Xinjiang University, Urumqi 830046, China; chjiahua@163.com (J.C.); bota119@126.com (B.B.); rabiya115@126.com (B.Y.); 2 Université de Bordeaux, IMS, Institut Polytechnique de Bordeaux, CNRS, UMR 5218, F-33607 Pessac CEDEX, France; mamatimin.abbas@ims-bordeaux.fr

**Keywords:** indole-3-butyric acid, polymer coating, molecularly imprinted polymer, fluorescence detection, molecularly imprinted polymer, sensor chip

## Abstract

In this paper, we report the preparation of molecularly imprinted polymer coatings on quartz chips for selective solid-phase microextraction and fluorescence sensing of the auxin, indole-3-butyric acid. The multiple copolymerization method was used to prepare polymer coatings on silylated quartz chips. The polymer preparation conditions (e.g., the solvent, monomer, and cross-linker) were investigated systemically to enhance the binding performance of the imprinted coatings. Direct solid-phase fluorescence measurements on the chips facilitated monitoring changes in coating performance. The average binding capacity of an imprinted polymer coated chip was approximately 152.9 µg, which was higher than that of a non-imprinted polymer coated chip (60.8 µg); the imprinted coatings showed the highest binding to IBA among the structural analogues, indicating that the coatings possess high selectivity toward the template molecule. The developed method was used for the determination of the auxin in mung bean extraction, and the recovery was found to be in the range of 91.5% to 97.5%, with an RSD (n = 3) of less than 7.4%. Thus, the present study provides a simple method for fabricating a fluorescent sensor chip for selective analysis.

## 1. Introduction

Phytohormones, which are typically present at low concentrations in plant tissues, regulate the growth and development of plants [1]. Auxins, an important group of phytohormones, were the first of the major plant hormones to be discovered. These hormones, including indole-3-acetic acid (IAA), indole-3-propionic acid (IPA), and indole-3-butyric acid (IBA), are involved in many aspects of plant growth and development [2]. IAA was the first plant hormone that was used to stimulate the rooting of cuttings. The second auxin, IBA, was discovered to also promote rooting and was determined to be even more effective than IAA [3,4]. Due to the higher stability of IBA versus that of IAA, both in solution and in plant tissue, IBA has a greater ability to promote adventitious root formation than does IAA.

The quantitative analysis of auxins is often required in agriculture and plant physiology. Various analytical techniques have been used for the analysis of auxins, mainly high performance liquid chromatography (HPLC) with different detectors such as mass spectrometer (MS) [5,6], ultraviolet spectrophotometer (UV) [7], fluorescence [8,9], and chemiluminescence [10]. Other techniques such as gas chromatography-mass spectrometer (GC-MS) [11,12], capillary electrophoresis (CE) [13], and enzyme linked immunosorbent assay (ELISA) [14] were also reported. Although these methods have been successfully applied for the sensitive analysis of auxins in a variety of plants, they suffer from limitations, such as the derivatization of auxins to methyl esters in GC, the high cost of equipment for a liquid chromatography-mass spectrometer (LC-MS), and the synthesis of the antibody for auxins and the cross-reactivity of antibodies in ELISA. Therefore, the development of a simple, rapid, inexpensive, and sensitive analytical method for determining auxins in complex matrices is still of critical importance.

Trace analyses of plant tissues and other complex matrices often rely on selective sample pre-treatment. Solid-phase microextraction (SPME) is a simple, time-efficient, and solvent-free sample pre-treatment technique based on the partitioning of analytes between the sample matrix and the polymer film coating on a substrate [15,16]. Recently, SPME approaches based on the use of highly selective, adsorbent molecularly imprinted polymers (MIPs) have been found to be attractive. MIPs are the only generic synthetic polymeric materials that possess properties like those of natural receptors [17,18]. MIPs are prepared by the co-polymerization of a cross-linker with functional monomers in the presence of a template molecule. After the removal of the target compound, binding sites are created in the polymer matrix that are capable of subsequent molecular recognition [19,20]. The MIPs can rebind selectively with the template and its analogous structures. To date, all of the SPME techniques performed manually or automatically have been used in combination with systems such as GC [21,22], HPLC [23], and CE [24]. In these approaches, the analyte is usually required to be desorbed from the coating by heating or dissolving for analysis.

**Figure 1:**
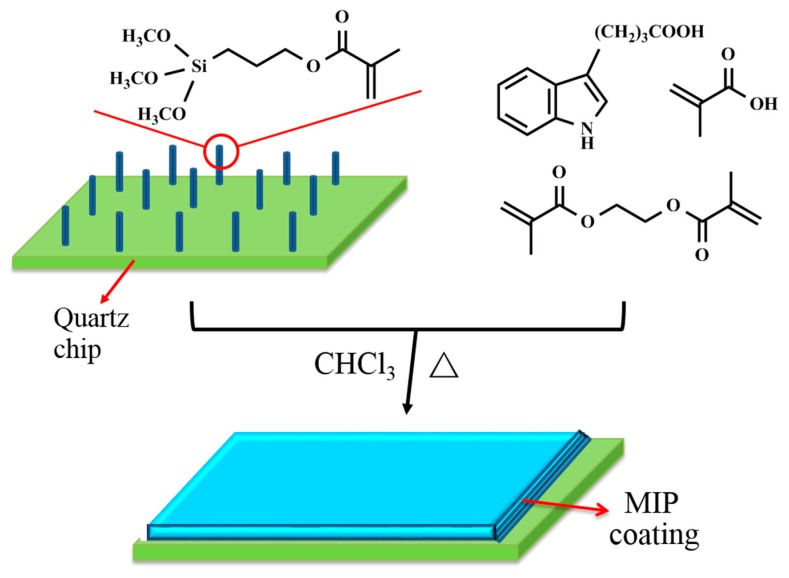
Schematic representation of MIP coating preparation.

The purpose of this work was to use selective MIPs as extraction coatings for the direct detection of auxins by their intrinsic fluorescence. Quartz chips were the appropriate substrate candidates for this purpose because modification of the silica surface has been intensively studied [25], and it can also facilitate UV and fluorescence detection. The MIP coatings with satisfactory thickness were prepared through the chemically attached silyl acrylate monomer on the quartz chips using the multiple co-polymerization method, shown in Figure 1.

The analyte was selectively retained on coatings and then quantified directly on the chip using a typical fluorescence spectrophotometer. Using this method, the high selectivity of the MIPs was combined with the high sensitivity of fluorescence detection. This combination has been observed to be favorable in previous studies [26]. We wish to report a simple method for fabricating a fluorescent sensor chip for selective analysis.

## 2. Materials and Methods

### 2.1. Chemical Reagents and Materials

Indole-3-acetic acid (IAA), indole-3-propionic acid (IPA), indole-3-butyric acid (IBA), tryptamine (TA), 3-(trimethoxysilyl)-propyl-methacrylate (MPS), vinylimidazole (VI), and methacrylic acid (MAA) were purchased from J&K Chemicals, Beijing, China. 4-Vinylpyridine (4VP) was purchased from Acros Organics, Geel, Belgium. Acrylic acid (AA), ethyl 2-(dimethyl amine) (DMEA), ethylene glycol dimethacrylate (EGDMA) and trimethylolpropane trimethacrylate (TRIM) were purchased from Tokyo Chemical Industry Co. Ltd (Tokyo, Japan). 2,2-Azobisisobutyronitrile (AIBN) was supplied by Shanghai Reagent Factory (Shanghai, China) and recrystallized in methanol. Acetonitrile, anhydrous ethanol, and disodium hydrogen phosphate (AR) were obtained from Sinopharm Chemical Reagent Co., Ltd, (Shanghai, China). Toluene and chloroform were obtained from Tianjin Chemical Reagent Factory (Tianjin, China). All the reagents listed above were of analytical grade. Double-distilled water and mung bean sprout were produced in the laboratory.

All fluorescence spectral measurements were conducted on a fluorescence spectrophotometer (CRT 970 Shanghai, China) with a tunable excitation light source. pH measurements were performed using a HANNA 211 pH meter (Milano, Italy). A DZF—6020 vacuum oven (Shanghai Yi Heng Technology Co. Ltd., Shanghai, China) was used for solidification. An 1810-B quartz automatic double water distiller was used for double distillation of deionized water (Shanghai, China).

### 2.2. Activation and Silylation of the Quartz Chip

Each quartz chip (1.3 cm, 2.6 cm) was washed with ethanol and water in an ultrasonic bath. Next, the substrate was treated in Piranha solution (a dangerously strong oxidizer), a 3:7 (*v*/*v*) mixture of H_2_O_2_ (30%) and H_2_SO_4_ (conc.), at 90 °C for 1 h [25]. Subsequently, the substrate was rinsed in distilled water, dried in N_2_, and then preserved in ethanol. The activated chip was then incubated in 2% (*v*/*v*) MPS solution, which was freshly prepared in dry toluene, under a nitrogen environment at 80 °C overnight. After washing with toluene and acetone, the substrate was dried at 100 °C for 4 h. The silylated chips were stored under dry N_2_.

### 2.3. Preparation of the MIP Coatings on the Quartz Chips

Each molecularly imprinted polymer-coated quartz chip was prepared according to the reported method, with minor modifications [27]. Briefly, 0.102 g of IBA (template) was placed in a glass vial and completely dissolved in 11.3 mL of chloroform (solvent), and then 0.172 g of MAA (monomer), 1.584 g of EGDMA (cross-linker), and 0.016 g of AIBN (initiator) were added. The prepolymer solution was sonicated for 10 min. Subsequently, a silylated chip was inserted into the solution and was deoxygenized with a stream of nitrogen for 10 min. The vial was sealed and placed in an oil bath for polymerization at 50 °C for 6 h. Next, the chip was pulled out carefully, resulting in a thin layer of the MIP coating on the quartz chip surface. This MIP-coated chip was placed in another empty vial filled with N_2_ and then heated to 60 °C for 24 h to cure the coating. The coated chip was eluted with a 10% (*v*/*v*) acetic acid methanol solution to remove the template until it could not be detected via a UV spectrophotometer. Five repetitions of the coating procedures were performed. A non-imprinted polymer (NIP)-coated chip was prepared simultaneously according to the same procedure described above but without adding the template (IBA).

### 2.4. Spectral Measurement

Each polymer-coated quartz chip was tested using a fluorescence spectrometer with the excitation light source set to a wavelength of 294 nm and the emission wavelength set to 370 nm. Steady-state emission spectra of the auxin on each coated chip were collected by placing the chip in the measurement cuvette at an angle of 90° relative to the excitation beam, as reported in our previous work [28]. This configuration can reduce the amount of excitation light going into the detector.

### 2.5. Adsorption Test

An IBA solution in 0.1M sodium phosphate buffer (pH = 2.5) of different initial concentrations (10–60 mg·L^−−1^) was used to test the adsorption of the coated chips. Six milliliters of the solution were added to glass vials, and the MIP- and NIP-coated chips were immersed into the respective vials. Next, the glass vials were shaken in a thermostatic oscillator for 12 h and then removed and washed with water. The adsorption quantities of the MIP- and NIP-coated chips were calculated according to the relative fluorescence intensity at 370 nm.

### 2.6. Sample Analysis

Auxin samples were extracted using the method described by Xi and co-workers, with some modifications [9]. Briefly, mung bean seeds were soaked in tap water and then germinated in trays. The water was replaced periodically every day. After 4–5 days, the sprouts were collected, accurately weighed, and then ground to powder. Five grams of shoot powder was placed in each glass bottle, and then 20 mL of 0.1M NaH_2_PO_4_-H_3_PO_4_ (pH = 9.0) buffer was added and maintained overnight at 4 °C in the dark. After 5 min of high-speed centrifugation, the supernatant was collected and extracted three times with an equal volume of petroleum ether. After adjusting the acidity of the supernatant solution to pH 2.5, the MIP-coated chip was immersed in 6.0 mL of the extraction solution for 4 h. After rinsing with buffer, the chip was examined by a fluorescence spectrometer as described in the Spectral Measurement Section 2.4, and the adsorption amount was calculated according to the relative fluorescence intensity.

## 3. Results and Discussion

The polymer performance was greatly dependent on the solvent, functional monomer, and cross-linking monomer used in the polymerization and the adsorption solvent, and these parameters were each optimized in the study.

### 3.1. Quartz Chip Silylation

Silylation of the chip surface is the key step to prepare stable coating. The chips were salinized using MPS. In silylation reaction, chip curing [29] has a very significant effect; in this study, the optimum level of curing was obtained at 100 °C over a period of 4 h. The silylation of each chip was demonstrated by the change in the water contact angle on the chip surface. Due to MPS silylation, contact angle of activated quartz chips changed from 13° to 114°. The change of the chip surfaces, from the hydrophilic to the hydrophobic, can prove that MPS was successfully grafted on the chip. Efficient surface copolymerization of the monomer and the cross-linker was ensured by the well silylation of the chip. Even after having been used 10 times, the IBA MIP coatings were found to retain good integrity, and no cracking was observed.

### 3.2. Selection of the Polymerisation Solvent

The amount of polymerization solvent used determines the morphology of the final polymer. With a low solvent content of approximately 50%, bulk polymerization occurs, whereas with a high solvent content (above 91%, usually approximately 98%), precipitation polymerization takes place.

In this study, the volume percentage of solvent was 86% of the volume of the polymer mixture, which is between the percentages that give rise to bulk polymerization and precipitation polymerization. In this case, the type of polymerization that is predominant depends on the polymerization time. Because polymerization for periods longer than 6 h results in a bulky and rather hard polymer and unsuccessful chip coating, we set the polymerization time to 6 h.

The type of polymerization solvent used determines the properties of the resulting polymer, such as surface polarity, porosity, and swelling. Acetonitrile and chloroform, the most commonly used solvents in the preparation of MIP, were selected as the polymerization solvents for investigation because the low-polarity organic solvents can improve the selectivity of MIP and because IBA exhibited good solubility in both solvents. The polymers to be investigated were prepared using MAA as a functional monomer and EGDMA as a cross-linker. The adsorption performance of the polymer coatings was investigated by incubating each chip in an IBA chloroform solution. After adsorption, the chip was rinsed with the solvent, and then the fluorescence intensity was directly measured by placing the chip in the measurement cuvette. The characteristic fluorescence spectra of IBA were observed on both the MIP and NIP chips. When the polymerization solvent used was chloroform and acetonitrile, the relative fluorescence intensity measured from the MIP chip was 86.10 and 32.94, respectively; this result indicates that the polymer formed in chloroform had a greater binding capacity. In our study, EGDMA-MAA polymers were observed to exhibit relatively stronger interaction via hydrogen bonding compared to the hydrophobic interaction and electrostatic interaction with IBA. This behavior was observed because chloroform is a better solvent for polymer and a better template to form stable hydrogen bonds than acetonitrile is.

### 3.3. Selection of Polymerization Monomers

**Figure 2:**
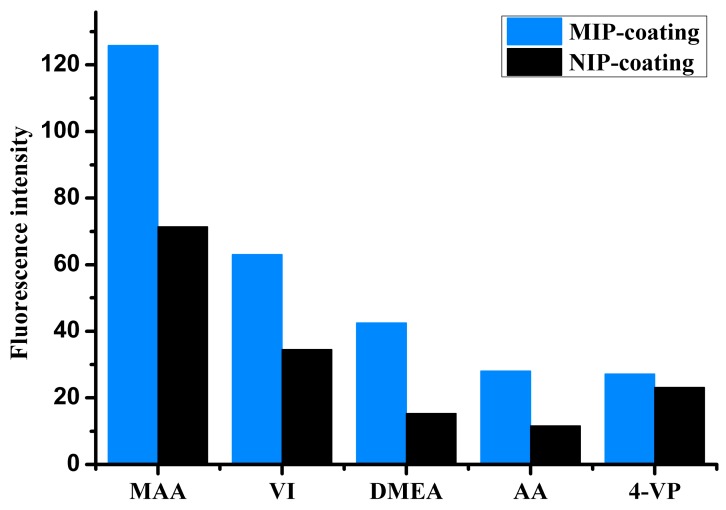
Chip fluorescence intensity for the different monomers used in the polymer coatings (5 mg·L^−1^ IBA solution in chloroform, λex/em = 294/370 nm).

Functional monomer is the key parameter in preparing MIP. In a previous study, we reported the NIP library approach for the screening of the best monomer [30]. The NIP library consisted of 18 cross-linked co-polymers synthesized from monomers commonly used in molecular imprinting. The NIP library, in the form of SPE cartridges, was investigated and successfully applied for water-compatible selective MIP preparation. In this study, a small NIP library was made in the form of polymer-coated chips. The NIP library consisted of five extensively applied monomers, namely MAA, 4-VP, VI, AA, and DMEA, for the preparation of five different MIP- and NIP-coated chips. The procedure described in 2.5 adsorption test was used for the monomer selection. As shown in Figure 2, IBA presented stronger adsorption on the all MIPs than corresponding NIPs. The order of relative strength of IBA adsorption on these MIPs was positively correlated to that of corresponding NIPs. This result is consistent with that reported by C. Baggiani and with the results of our previous studies [30,31]. C. Baggiani et al. reported that there is a clear and positive correlation between the apparent affinity constants measured for the NIP and MIP libraries. Among the polymer coatings studied here, the MAA-based coating was found to exhibit the highest change in relative fluorescence intensity due to IBA adsorption, which indicates that the polymer shared the strongest binding interaction with the target molecule. This was probably because hydrogen bonding is the major type of interaction between IBA and the functional monomers in the polymer, and MAA has the strongest hydrogen bonding interaction with IBA in the monomers tested in this study. As shown in Figure 2, the MAA-based NIP coating exhibited strong binding to IBA. In the MIP coating, IBA and MAA could form a complex in the polymer mixture, resulting in a three-dimensional cross-linked structure via copolymerization. After elution of the template, a predetermined complementary binding site remained in the polymer coating.

The role of a cross-linker in an imprinted polymeric network is to secure the functional groups of the functional monomers in specific locations and directions around the template molecules, thereby preserving the structure of the binding sites (cavities). The recognition ability of molecularly imprinted polymers and their physical and chemical properties strongly depend on the degree of cross-linking and on the nature of the cross-linker [32]. In this study, the most commonly used cross-linking agents (DVB, EGDMA, and TRIM) were selected for the preparation of three different MIP- and NIP-coated chips using MAA as the functional monomer and chloroform as the polymerization solvent. Because relatively strong background fluorescence was observed on the DVB-based polymer, only the other two cross-linkers were investigated further.

After IBA binding, the relative fluorescence intensity (141.04) of EGDMA based MIP coating was higher than that (92.33) of TRIM-based MIP coating, thus EGDMA was selected and used for the rest of the study.

### 3.4. Coating Characterization

The chemical structure of the MIP coating could be confirmed to be the copolymerization product of the monomer and the crosslinker using an infrared spectroscopy study (see Figure S1 in the Supplementary Materials). NIP and MIP coating before and after washing present quite similar infrared spectra. The characteristic infrared absorption peaks were found at 2954–2963 cm^−1^ (methyl groups), 1724–1728 cm^−1^ (carbonyl groups), and 1387–1389 cm^−1^ (methyl groups). Significantly, the minor peaks at 1637-1655cm-1, which were attributed to the residual C=C bond, were found for all the coatings. Absorption peaks were observed at 3308 cm^−1^ attributed to hydroxyl group of the MAA or/and template IBA in MIP coating. Before washing, MIP coating showed clear absorbance at 1537 cm^−1^, which is attributed to IBA. After washing, the band had disappeared. That is the clear evidence of complete elution of the imprinted molecule from the imprinted sites. The intense absorption peak at 1102 cm^−1^ is attributed to siloxane bond (Si-O-Si) stretching that constitutes the structural skeleton of the quartz chip. Scanning electron micrographs presenting the morphological structures of IBA MIP-coated chip are shown in Figure S2 (in the Supplementary Materials), respectively. The results demonstrated that a homogeneous surface was obtained for coatings of controlled thickness with the multiple coating procedures shown in Figure S3 (in the Supplementary Materials).

### 3.5. Selection of Adsorption Solvent

**Figure 3:**
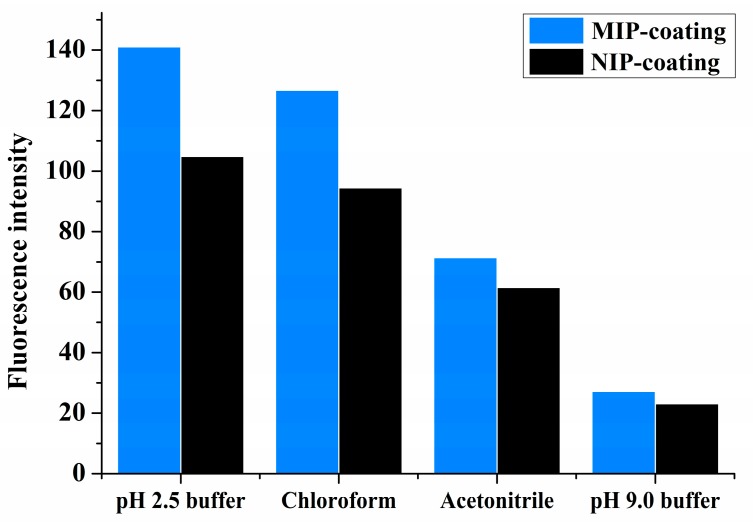
Fluorescence intensities of MIP- and NIP-coated quartz chips in different solutions (5 mg·L^−1^ IBA solution, λex/em = 294/370 nm).

The choice of solvent is important for the template–MIP interactions. Solvents of different polarities were investigated, as shown in Figure 3. In all solvents—i.e., chloroform, acetonitrile, and pH 2.5 and pH 9.0 of 0.1M sodium phosphate buffer solutions—IBA showed better affinity on the MIPs than on the NIPs. Nonpolar or acidic buffer solvents were observed to be better for IBA adsorption on the polymers. This result once again indicates that hydrogen bonding is the dominant interaction in the specific binding of the MIP system herein described. The highest adsorption (increase in the fluorescence intensity) was observed in a buffer of pH 2.5. In addition, the binding difference of IBA between the MIPs and NIPs was the most pronounced in this solvent, followed by the binding difference in chloroform. Because adsorption capacity is a very important factor in surface imprinting, the buffer solution is also favored for chip coating in sample analysis.

### 3.6. SPME Performance

**Figure 4:**
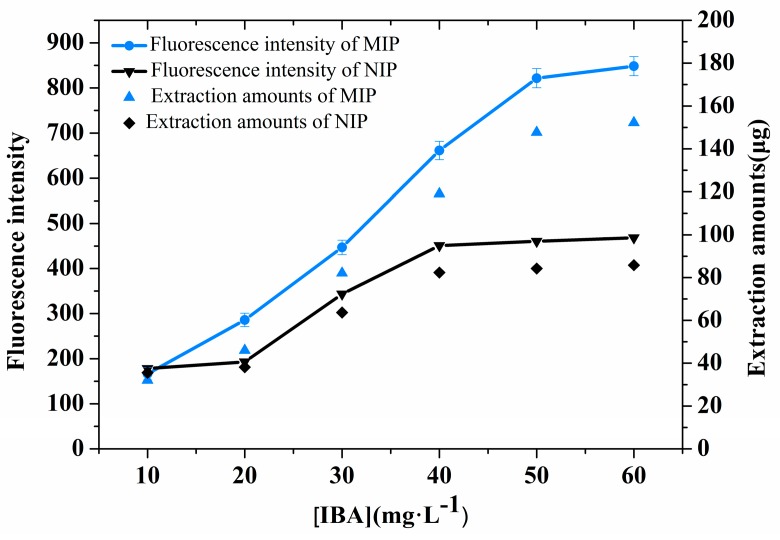
Curve showing the amount of IBA extracted by the polymer coating at pH 2.5.

The extraction performances of the MIP- and NIP-coated chips were investigated using IBA solutions of different initial concentrations (10–60 mg·L^−1^) at pH 2.5. Figure 4 shows that the extraction amount on the MIP and NIP coatings increased with the IBA concentration and that the adsorption amounts of the two coatings were almost saturated at 50 mg·L^−1^. The extraction capacities of the MIP- and NIP-coated chips were 152.9 µg and 60.8 µg, respectively. Clearly, the extraction amounts of the MIP-coated chips were much higher than those of the NIP-coated chips. This result indicates that the MIP-coated chips exhibit high selectivity and specific adsorption capacity toward the template IBA. The MIP coating exhibited a quantitative response towards IBA (Figure 4) in the buffer, and a quantitative correlation was determined, as described by the equation

FI = 16.80 × [IBA] − 27.09,(1)

where FI denotes the emission intensity of the chip after IBA adsorption, and [IBA] represents the concentration (mg·L^−1^) of IBA in the buffer solution. It should be noted that the FI value can be linearly (R^2^ = 0.9918) plotted against the IBA concentration in solution over the range of 2 to 50 mg·L^−1^, indicating that the MIP-coated chip can be used to quantitatively determine IBA over this concentration range.

### 3.7. Selectivity Test

**Figure 5:**
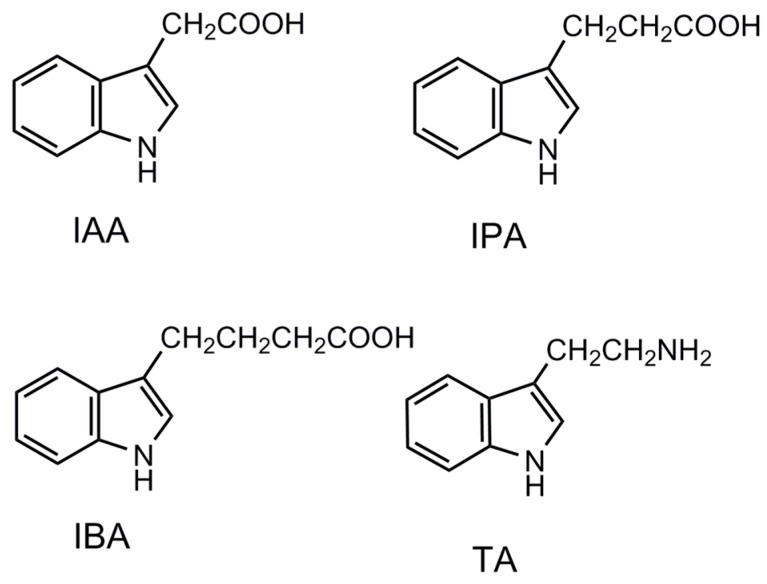
Molecular structures of IBA and its analogues IAA, IPA, and TA.

**Figure 6:**
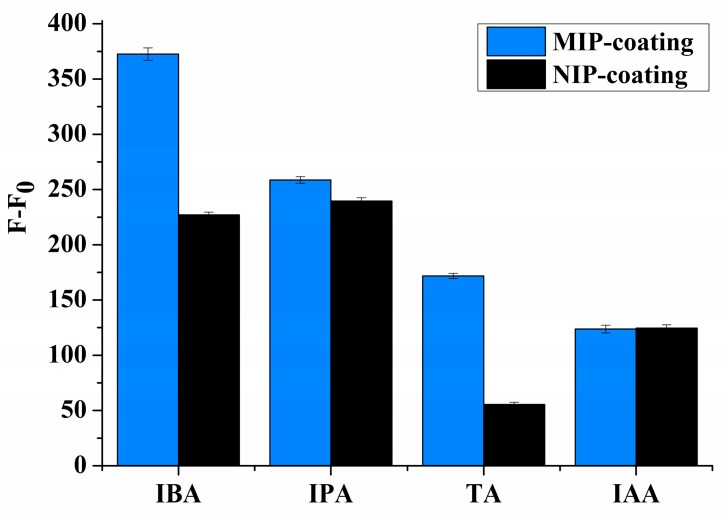
Fluorescence intensities of IAA, IPA, and TA adsorbed on the MIP/NIP coating (10 mg·L^−1^ target solutions in buffer pH = 2.5, λex/em = 294/370 nm).

The origins of molecular recognition in MIPs are generally believed to arise from pre-organization of functional groups within a shape selective cavity [33] The molecular recognition of MIP- and NIP-coated chips were examined using three substituted indoles as structure analogues—namely, IAA, IPA, and TA (see the corresponding molecular structures in Figure 5). The binding of IBA on MIP was higher than that of all the other indole compounds (see Figure 6). All the compounds tested in this study exhibited stronger binding on the MIPs than on the NIPs. On the IBA-MIP-coated chips, the extraction amounts of IBA, IPA, TA, and IAA were 31.9, 15.2, 13.3, and 8.6 µg, respectively, proving that even with high similarity between the homologues (IAA, IPA, and IBA) and the template, the imprinting was sufficient for the discrimination of the different compounds. As the homologues concerned, the template IBA was found to have the highest binding on MIPs, followed by IPA and then IAA, which shows excellent shape selectivity of the MIPs. TA is identical to IPA in structure but features an amino group instead of a carboxyl group. TA had lower binding on the MIPs than IPA did. This evidence suggests that MIPs have functional group selective recognition as well. NIPs showed the lowest binding to TA, but could not discriminate the homologues according to the shape of the template (see Figure 6). This indicates that there was only functional group selective recognition in NIPs. We propose that the shape selectivity appears to be the dominant mechanism for selectivity by the imprinting effect.

### 3.8. Sample Analysis

The MIP coating was used to selectively extract IBA from mung bean samples. The solvent extraction process is necessary to transfer the analyte from the sample into a liquid phase. In this study, phosphate buffer with pH 9.0 was used as the extraction solvent. To reduce the matrix effect on the following measurement, petroleum ether was applied to remove some sources of interference. To achieve optimum uptake by the MIP coating, the pH of the aqueous solution was adjusted to 2.5 prior to extraction. The fluorescence intensity of the IBA-MIP-coated chip was measured using a fluorescence spectrophotometer after simply rinsing the chip with the phosphate buffer of pH 2.5. The amount of auxin observed in the mung bean was 0.95 µg·g^−^^1^, as calculated using a calibration equation. To further evaluate the extraction performance of the MIP-coated chip, spiked samples were tested. The extraction recoveries were observed to be 91.5% (RSD = 6.1) and 97.5% (RSD = 7.4), with n = 3 for the spiked concentrations of 0.8 µg·g^−1^ and 2 µg·g^−1^, respectively. Thus, the present study provides a simple method based on a MIP-coated chip that can be applied for the selective and sensitive determination of auxin in complex samples. To realize automatic analysis and to minimize the sample volume required for measurement, the construction of an on-line detection apparatus using the chip as a flow cell is in progress.

## 4. Conclusions

In summary, we developed a fluorescent sensor chip based on molecular imprinting technique, using indole-3-butyric acid as the model system. The system is found to be simple in the terms of fabrication and application. Multiple bulk copolymerization methods were applied for the preparation of imprinted polymer coatings with appropriate thickness on the surface of silylated quartz chips. The polymer coatings are selective enough to discriminate the template molecular from its homologues in aqueous buffer. A good linear relationship was established between the concentrations of IBA versus the fluorescence intensity of the auxin extracted on the MIP coating. The MIP-coated chip was successfully applied for the analysis of auxin in mung bean sprouts with high accuracy. These features demonstrated that the chip has great potential for practical applications. To realize automatic analysis and to minimize the sample volume required for measurement, the construction of an on-line detection apparatus using the chip as a flow cell is in progress.

## Supplementary Materials

The following are available online at http://www.mdpi.com/1424-8220/17/9/1954/s1, Figure S1: ATR-FTIR spectra of SiO_2_@NIP; SiO_2_@MIP after wash; SiO_2_@MIP before wash, Figure S2: Scanning electron microscope image, and Figure S3 The relationship between times of MIP coating and coating thickness.

## Abbreviations

AA	acrylic acid
AIBN	2,2-azobisisobutyronitrile
DVB	divinylbenzene
DMEA	ethyl 2-(dimethyl amine)
EGDMA	ethylene glycol dimethacrylate
IAA	indole-3-acetic acid
IBA	indole-3-butyric acid
IPA	indole-3-propionic acid
MAA	methacrylic acid
MIP	molecularly imprinted polymer
MPS	3-(trimethoxysilyl)-propyl-methacrylate
NIP	non-imprinted polymer
SPME	solid-phase microextraction
TA	tryptamine
TRIM	trimethylolpropane trimethacrylate
VI	vinylimidazole
4VP	4-vinylpyridine
HPLC	high performance liquid chromatography
MS	mass spectrometer
UV	ultraviolet spectrophotometer
GC-MS	gas chromatography-mass spectrometer
CE	capillary electrophoresis
ELISA	enzyme linked immunosorbent assay
LC-MS	liquid chromatography-mass spectrometer
