# Supplementary material for: A Simple and Selective Fluorescent Sensor Chip for Indole-3-Butyric Acid in Mung Bean Sprouts Based on Molecularly Imprinted Polymer Coatings
Source: Sensors (Basel). 2017 Aug 24;17(9):1954. doi: 10.3390/s17091954 (PMC5620659; doi:10.3390/s17091954)
Supplement: Supplementary file 1 [file sensors-17-01954-s001.pdf]

A simple and selective fluorescent sensor chip for indole-3-butyric acid in mung bean sprouts based on molecularly imprinted polymer coatings

Jiahua Chang [1], Bota Bahethan [1], Turghun Muhammad [1,*], Burabiye Yakup [1] and Mamatimin Abbas [2]

[1] College of Chemistry & Chemical Engineering, Xinjiang University, Xinjiang Key laboratory of Oil and Gas Fine Chemicals, Urumqi 830046, People's Republic of China

[2] Université de Bordeaux, CNRS, IMS, UMR 5218, F-33400 Talence, France

* Corresponding author: *Dr. Turghun Muhammad, College of Chemistry & Chemical Engineering, Xinjiang University, Urumqi 830046, People's Republic of China Tel.:+86 9918582564; Fax.: +86 9918582809; Email:* turghunm@sina.com

Supporting information

Figure S1

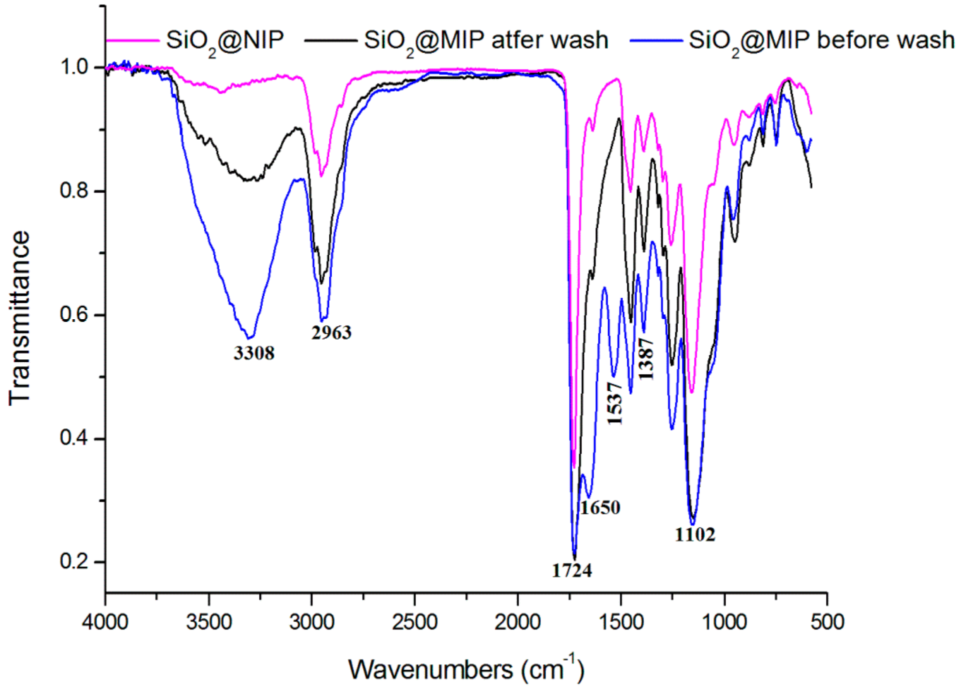

Figure.S1. ATR-FTIR spectra of SiO_2@NIP; SiO_2@MIP after wash; SiO_2@MIP before wash

NIP coating, MIP coating before and after wash presented quite similar infrared spectra, as shown in Fig.S1. Before washing, MIP coating showed clear absorbance at 1537 cm^{-1}, which is attributed to IBA. After washing, the band is disappeared. That is the clear evidence of complete elution of the imprinted molecule from the imprinted sites.

Figure S2

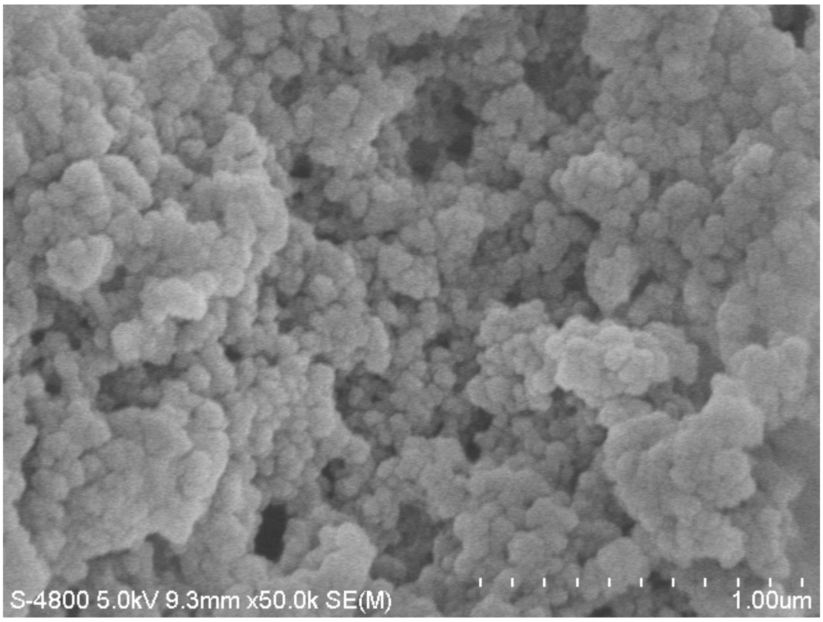

Fig. S2(a). Scanning electron microscope image of surface of the MIP coated chip

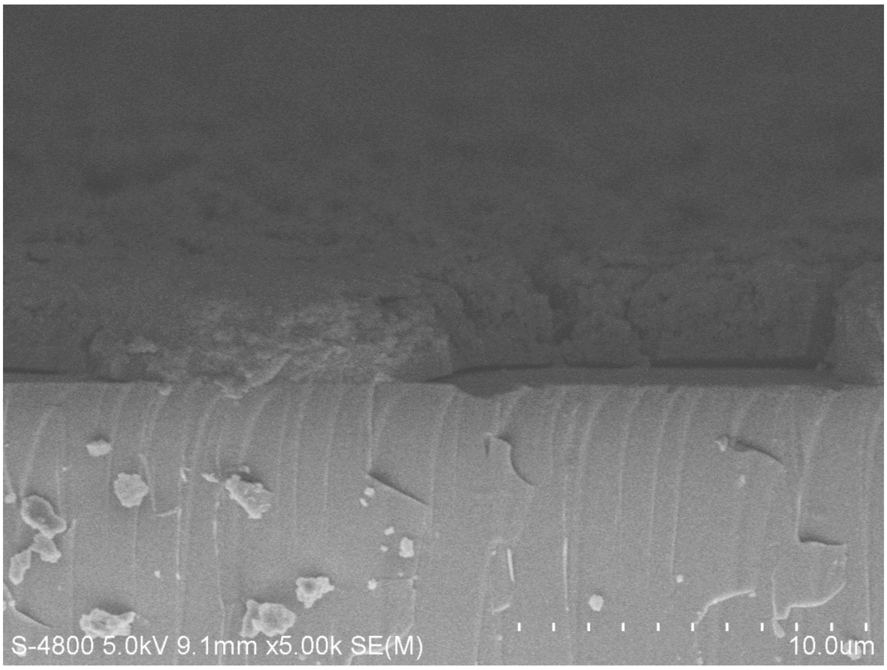

Fig. S2(b)Scanning electron microscope image of side of the MIP coated chip cut view

Figure S3

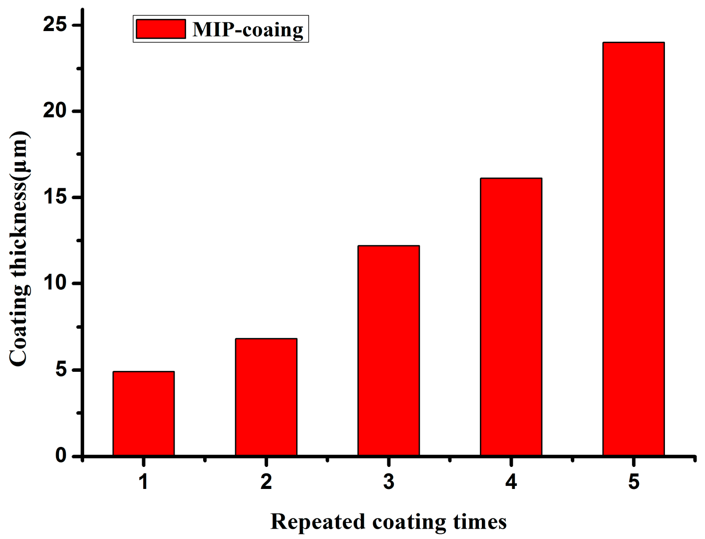

Fig.S3. The relationship between times of MIP coating and coating thickness
